# Imipenem Resistant *Pseudomonas aeruginosa*: The fall of the final quarterback

**DOI:** 10.12669/pjms.313.7372

**Published:** 2015

**Authors:** Nadya Ameen, Zahida Memon, Shehla Shaheen, Ghulam Fatima, Farah Ahmed

**Affiliations:** 1 Nadya Ameen, MBBS. Department of Pharmacology, Ziauddin University, St-4B, Sharah-e-Ghalib Block6, Karachi-75600, Pakistan; 2 Zahida Memon, MBBS, M.Phil, PhD. Department of Pharmacology, Ziauddin University, St-4B, Sharah-e-Ghalib Block6, Karachi-75600, Pakistan; 3 Shehla Shaheen, MBBS, M.Phil. Department of Pharmacology, Ziauddin University, St-4B, Sharah-e-Ghalib Block6, Karachi-75600, Pakistan; 4 Ghulam Fatima, MBBS, MCPS, M.Phil Central Lab, Civil Hospital, Karachi, Pakistan; 5 Farah Ahmed, MBBS, MPH. Department of Community Health Sciences, Ziauddin University, Karachi, Pakistan

**Keywords:** Imipenem-resistant *Pseudomonas aeruginosa* (IRPA), Metallo-beta-lactamases (MBL), combined disk test

## Abstract

**Objective:**

To isolate, determine the frequency, and study the demographic trends of MBL positive *Pseudomonas aeruginosa* from imipenem resistant isolates collected from clinical samples in a tertiary care hospital of Pakistan.

**Methods:**

In this cross sectional study a total of 230 strains of Pseudomonas were isolated from various clinical specimens on the basis of culture and biochemical tests. Imipenem resistant isolates were selected by Kirby Bauer Diffusion technique, followed by screening for MBL production by Imipenem EDTA Combined Disk Test. Demographic details of each patient were recorded on a separate questionnaire. Chi-Square goodness-of-fit test was computed to review the isolation of MBL positive isolates (P-value ≤ 0.05) in different specimen.

**Results:**

Out of 230 strains of *P. aeruginosa* 49.5% were imipenem resistant; MBL production was confirmed in 64.9% of the resistant isolates. Resistance to polymyxin B (12.5%) was notable. Majority of the MBL positive strains were isolated from patients aged between 20-39 years (45.9%) and the predominant source was pus (43.24%) which was found to be statistically significant (P-value=0.04). Outpatient departments (24.3%) and burn unit (21.6%) were the major places for resistant isolates.

**Conclusion:**

MBL production is one of the major causes of IRPA. Increasing resistance to polymyxin B is grave. Due to acquisition of MBL strains MDR *P. aeruginosa* has become endemic in tertiary setups.

## INTRODUCTION

The notorious Metallo-beta lactamases belong to Ambler molecular class B carbapenemases. Metallo-beta-lactamases are able to hydrolyze virtually all beta-lactam antibiotics including carbapenems. They are not even inhibited by beta-lactamase inhibitors. Since the last decade MBL producing isolates have especially appeared in *P. aeruginosa*.^1^,^2^ Infact thirty years ago medical students did not study Pseudomonas aeruginosa as a serious pathogen, but today cosmopolitan multidrug resistant Pseudomonas aeruginosa is playing havoc with medical therapeutics.^3^ *P. aeruginosa* is amongst the commonest Gram negative nosocomial pathogen with 50% mortality rate in systemic infections. It is difficult to treat as the bacteria acquires additional modes of resistance such as acquisition of metallo-beta-lactamases (MBL) genes even during the course of therapy.^4^ This makes *P. aeruginosa* virtually invincible against our best reserve, imipenem^5^ employed in treatment of multi drug resistant strains.^6^ To complicate matters, imipenem resistant strains are often resistant to other antibiotic groups and worsen the prognosis in terms of mortality, morbidity and expenditure.^2^,^7^ Thus prompt identification of MBL strains is pertinent.

Globally, MBL strains of Pseudomonas have been detected in the subcontinent,^8^ Middle East^9^,^10^ and various countries worldwide.^11^ In Pakistan, research has been done on diagnostic techniques for prompt identification of MBL strains in Gram negative organisms.^12^,^13^ Few studies have reported 78%-100% production of MBL in carbapenem-resistant *P. aeruginosa*.^14^,^15^

The ubiquitous nature of the bacteria and irrational use of antibiotics via prescribers, dispensers and patients provide a fertile ground for dissemination of multidrug resistant strains in our setup.^16^ Therefore the current study was designed to determine the frequency and demographic trends of MBL positive strains of IRPA isolates in different clinical specimen of a tertiary care hospital of Pakistan.

## METHODS

### Study period and sampling

This cross sectional descriptive study was conducted from October 2013 to September 2014 at Civil Hospital Karachi. Samples were submitted to Central Lab for culture and sensitivity from indoor patients and outpatient clinics. Routine specimens were taken including pus, wound swab, blood and endotracheal tube secretions.

### Culture

These samples were processed as per microbiological procedures (CLSI Guideline 2013).^17^ After a written informed consent detailed information of the patients and isolates was recorded on a separate questionnaire.

### Biochemical identification of P. aeruginosa

All non-lactose fermenting colonies on MacConkey Agar were picked up for Gram’s stain and biochemical identification, such as motility, pigment production, citrate, catalase and oxidase test.

### Antimicrobial susceptibility testing

Antimicrobial sensitivity was performed on Mueller Hinton Agar plates by Kirby-Bauer disk diffusion technique. Following antibiotic disks (Oxoid. UK) were used cefoperazone/sulbactam (SCF) 105µg, cefepime (FEP) 30 µg, ceftazidime (CAZ) 30µg, imipenem (IPM) 10µg, piperacillin/tazobactam (TZP) 100µg/10µg, aztreonam (ATM) 30µg, amikacin (AK) 30µg, tobramycin (TOB) 10µg, gentamicin (CN) 10µg, ciprofloxacin (CIP) 5µg, polymyxin B (PB) 300 units.

### Detection of Metallo-beta-lactamase by imipenem EDTA (ethylenediaminetetraacetic acid) combined disk test

Only those strains of *P. aeruginosa* which were imipenem resistant by Kirby-Bauer disk diffusion method underwent detection for MBL production. Imipenem EDTA combined disk test was carried out according to Yong et al.^18^

Statistical analysis was done on SPSS Version 20. Descriptive statistics was carried out for both numerical and categorical data. Mean and standard deviation represent numerical data while frequency and percentage represent categorical data. Chi-square goodness of fit test was computed to review the isolation of MBL positive isolate considering P-value ≤ 0.05 is statistically significant. Bar charts were used to represent the resistance pattern of MBL positive isolates, while graphical presentation of frequency of MBL positive isolates in different departments was done by pie chart.

## RESULTS

**Fig. 1:**
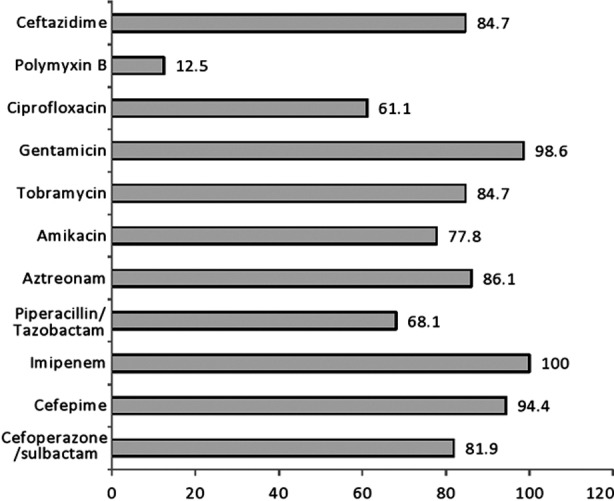
Resistance pattern of MBL positive isolates.

A total of 230 strains of Pseudomonas were isolated out of which, 114 (49.5%) were imipenem resistant by Kirby Bauer Disk Diffusion Technique. Amongst these 114 resistant isolates MBL production was confirmed in 74 (64.9%) strains by imipenem EDTA combined disk test. The least resistance was observed against polymyxin B (12.5%). The resistance pattern for the remaining therapeutic agents was between 61.1% and 98.6% (Fig.1).

The mean age of patients in which MBL producing strains were isolated was 28.2 ±18years (range 1month to 85 years). Majority of the cases were isolated in age group of 20-39 years, 34(45.9%) and the least in patients sixty years or above 6 (8.1%) patients (Table-I).

**Table-I:** Age distribution of MBL positive strains (n=74).

Age in years	MBL positive strains n (%)
1 month to19 years	23 (31.1%)
20-39 years	34 (45.9%)
40-59 years	11(14.9%)
≥ 60 years	6 (8.1%)

**Fig.2:**
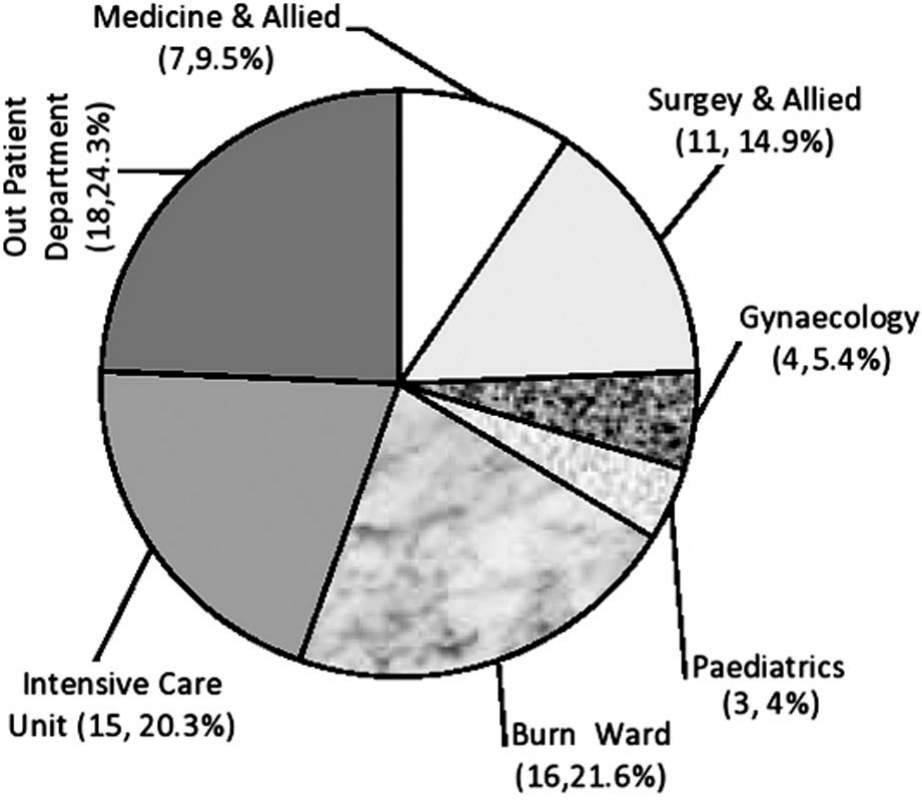
Frequency of MBL positive strains in different departments.

Gender wise distribution of MBL positive isolates showed that 42 (56.8%) were males and 32 (43.2%) were females. Out of 74 MBL positive isolates majority of cases were obtained from OPD, 18 (24.3%). Amongst wards the highest number of MBL positive isolates was recorded from burn ward, 16 (21.6%) and the least in paediatrics, 3 (4%). (Fig.2)

The predominant source of MBL positive strain was pus, 32 (43.29%) followed by wound swabs 16 (21.6%) and the least was in endotracheal tube secretions 12 (16.2%). Chi-square goodness-of-fit test was applied which showed that isolation of MBL positive isolate from pus was statistically significant (χ^2^ (3) = 13.58, P-value= 0.004) (Table-II).

**Table-II:** Isolation of MBL positive strains from different specimen (n=74).

Specimen	No. of isolates n (%)	P-value
Pus	32 (43.24%)	0.004
Wound Swab	16 (21.62%)	
Blood	14 (18.91%)	
Ett secretions	12 (16.21%)	

## DISCUSSION

Imipenem is a potent cell wall synthesis inhibitor. Like all beta-lactam antibiotics it produces its therapeutic effects by crossing the cell wall through porins and binding to penicillin binding proteins (PBP) in the cell membrane.^19^ In *P. aeruginosa* porin OprD mutation in conjunction with production of AmpC^6^ or acquisition of MBL genes by the organism causes resistance to imipenem.^20^

In our study the frequency of MBL positive strains in IRPA was 64.9%; whereas in studies conducted in Pakistan, India and Brazil all IRPA strains were MBL positive.^11^,^14^,^21^ In this regard, Nasrin et al. has reported 43% imipenem resistant MBL strains in a tertiary care hospital of Bangladesh.^22^ All these figures suggest that MBL strains have the propensity to become endemic in a hospital.

In this study MBL positive isolates were predominant in males 42 (56.8%) than females 32 (43.2%). This is similar to an Indian study done by Ranjan et al.^23^ In comparison of different age groups, the majority of the MBL positive isolates were identified in patients in age group of 20-39 years, 34 (45.9%) and least in patients sixty years or above, 6 (8.1%) as shown in Table-I. This is in contrast to a study conducted by Bashir et al. where majority of the resistant isolates were above sixty years, 17 (51.5%).^2^ This contrast exists probably because in our country more patients in this age group (20-39 years) are being admitted to hospitals, from where they are acquiring resistant pathogens due to improper sterilization techniques and irrational use of antibiotics.

In our study highest number of MBL positive IRPA isolates were isolated from pus, 32 (43.29%), followed by wound swab 16 (21.6%). In contrast, Bashir et al.^2^ reported least in pus, 2 (6.1%). This is because in our setup majority of the patients had postoperative wound complications or burns. They remained in the ward for a longer duration. Inadequate antiseptic measures and poor hygiene in wards contributed in acquiring the resistant strains.

Among all 74 MBL positive isolates, 18 (24.32%) were detected in OPD patients. This is in contrast to Bashir et al.’s study in which all the isolates were obtained from hospitalized patients.^2^ This shows that in our country eventually MBL producing strains have become endemic.

Our study showed that the major contribution of MBL strains was from burn ward, 16 (21.6%) followed by ICU, 15 (20.3%) and Surgical and Allied Departments 11(14.9%) whereas Bashir et al. and Nasrin et al. has linked ICU significantly to be the major source of MBL strains.^2^,^22^

In 2005 Naqvi et al. observed that although MDR Pseudomonas was highly prevalent in burn ward at JPMC it was 77.3% sensitive to imipenem.^24^ Paradoxically our core finding was that imipenem resistance is increasing in burn patients.

Currently the artillery available against *Pseudomonas aeruginosa* include antipseudomonal penicillins (ticarcillin and piperacillin), third and fourth generation cephalosporins (ceftazidime, cefoperazone and cefepime), carbapenems (imipenem and meropenem), aminoglycosides (amikacin and tobramycin) and fluroquinolones (ciprofloxacin). However, due to acquisition of MBL strains *P. aeruginosa* has finally outsmarted our best treatment options. Like other studies our research has also demonstrated high resistance against all beta-lactam antibiotics. As we know by definition MBL strains are sensitive to aztreonam versus all other beta-lactams. In contrast in our study there was 86.1% resistance against aztreonam whilst an Indian study showed 100% resistance.^25^

Imipenem is still being used as empirical therapy in the treatment of MDR pathogens. However, due to resistance against this wonder drug, Polymyxin B a basic peptide with a narrow therapeutic index is being used as an alternate treatment.^26^ Though our study has revealed an emerging resistance for polymyxin B (12.5%) many countries with a high frequency of MBL positive strains have 100% sensitivity for polymyxin B.^2^,^27^

## CONCLUSION

IRPA is increasing in tertiary care setups and acquistion of MBL strains is one of the major cause of resistance to this wonder drug. Emergence of polymyxin B resistant strains may push us back into a pre-antibiotic era. Since MBL strains have a propensity to disseminate globally, it is feared that MDR *P. aeruginosa* may soon become a pandemic pan resistant strain. The role of bacteriophage therapy in MDR strains is still in embryonic phase^28^; and we are far from developing molecules like MBL inhibitors there is an immediate urge to circumvent this increasing resistance to carbapenems. This can be achieved by limiting the usage of imipenem to MDR strains and avoiding irrational prescription writing.
